# Thrombosis and Risk Factors: A Comment

**DOI:** 10.5505/tjh.2012.837131

**Published:** 2012-03-05

**Authors:** Nejat Akar

**Affiliations:** 1 TOBB Economy and Technology University Hospital, Ankara, Turkey

## REPLY

**Thrombosis and Risk Factors**

I read the comment of Toprak et al. on my letter tothe Editor appeared in a recent issue of the journal withgreat interest [1]. They pointed a missing point in my letterand then focused on the effect of folate metabolism onhomocysteine and metihelenetetrahydrofolate gene polymorphismat 677 C to T extensively [1]

I would like to express my thanks to Toprak et al. givinga chance to explain my view on this matter once more.

As it is well known that there is a continuing debateon homocysteine metabolism, MTHFR SNP’s and folatemetabolism and there are several published reviews onthis subject not reaching to a conclusion.

Recently we reported that MTHFR 677 T has an influenceon Hcy levels in Turkish population. But also wefound another possible MTHFR gene haplotype, whichdoes not have an effect on Hcy levels [2].

Furthermore, there are also rare novel SNPs publishedwithin the MTHFR 677 region with an allele frequencyof 1 in 3000-4000 sample, including MTHFR 678 C-A(Ala222Ala) in Turkish population [3,4,5] which may leadto erroneous technical reporting.

Since our first publication on homocysteine relatedgene polymorphisms in Turkish population in 1998 [6], and the others following the first paper, I reached to aconclusion that without determining homocysteine levels,analyzing MTHFR 677C-T solely at the DNA levelis unnecessary, not cost effective and does not have anyclinical value especially in Turkish population.

So, only homocysteine levels should be routinely analyzedand not the MTHFR 677 T SNP alone. If any causeof high homocysteine levels could not be found, thenMTHFR 677 T analysis can be performed.
